# Integrating Microtissues in Nanofiber Scaffolds for Regenerative Nanomedicine

**DOI:** 10.3390/ma8105342

**Published:** 2015-10-09

**Authors:** Laetitia Keller, Quentin Wagner, Damien Offner, Sandy Eap, Anne-Marie Musset, Manuel Arruebo, Jens M. Kelm, Pascale Schwinté, Nadia Benkirane-Jessel

**Affiliations:** 1 INSERM (French National Institute of Health and Medical Research), “Osteoarticular and Dental Regenerative Nanomedicine” laboratory, UMR 1109, Faculté de Médecine, Strasbourg Cedex F-67085, France; lkeller@unistra.fr (L.K.); wagner.quentin@gmail.com (Q.W.); doffner@unistra.fr (D.O.); sandyeap@yahoo.fr (S.E.); musset@unistra.fr (A.-M.M.); 2 Université de Strasbourg, Faculté de Chirurgie Dentaire, 1 place de l’Hôpital, Strasbourg F-67000, France; 3 Hôpitaux Universitaires de Strasbourg (HUS), Strasbourg F-67000, France; 4 Department of Chemical Engineering and Aragon Nanoscience Institute, University of Zaragoza, C/Mariano Esquillor, s/n, Zaragoza 50018, Spain; arruebom@unizar.es; 5 InSphero AG, Wagistrasse 27, Schlieren 8952, Switzerland; jens.kelm@insphero.com

**Keywords:** nanofibrous implant, microtissues, regenerative nanomedicine, bone regeneration

## Abstract

A new generation of biomaterials focus on smart materials incorporating cells. Here, we describe a novel generation of synthetic nanofibrous implant functionalized with living microtissues for regenerative nanomedicine. The strategy designed here enhances the effectiveness of therapeutic implants compared to current approaches used in the clinic today based on single cells added to the implant.

## 1. Introduction

Bone regeneration is a complex, well-orchestrated physiological process, which occurs during normal healing of fractures, and is involved in continuous remodeling throughout adult life. In the clinic, bone regeneration can be required in large quantities [1], such as for skeletal reconstruction of large bone defects resulting from trauma, tumor resection or cases in which the regenerative process is compromised (non-unions, osteoporosis). Current processes promoting bone-regeneration, including the free fibula vascularized graft, autologous bone graft, allograft implantation, and use of growth factors are unsatisfactory as they induce insufficient quantities of bone [2]. Current cell-based tissue engineering techniques often involve the use of 3D scaffold materials with appropriate mechanical and structural properties, to trigger the regenerative response of cells. Such scaffolds have been developed through various fabrication techniques [3] (electrospinning, microfabrication, lithography, *etc.*).

The key for a clinically transferable tissue engineering approach is the fast maturation of the tissue throughout the whole scaffold to support healing processes as shown for the bone, or to replace dysfunctional tissue. Most 3D models combine a small number of cells with a large amount of scaffold (natural or synthetic), maximizing cell-to-scaffold interactions, but in most *in vivo* tissues, cell-to-cell interactions are the most important [4]. The use of cell microtissues without external extracellular matrix is largely used for drug screening however, for regenerative medicine, single cells combined with scaffolds are still the predominant dogma.

Here we report a novel approach in bone tissue engineering leading to bone induction, combining nanofibrous poly(ε-caprolactone) (PCL) scaffolds [5,6] together with preformed tridimensional bone microtissues from osteoblasts.

## 2. Results and Discussion

**Figure 1:**
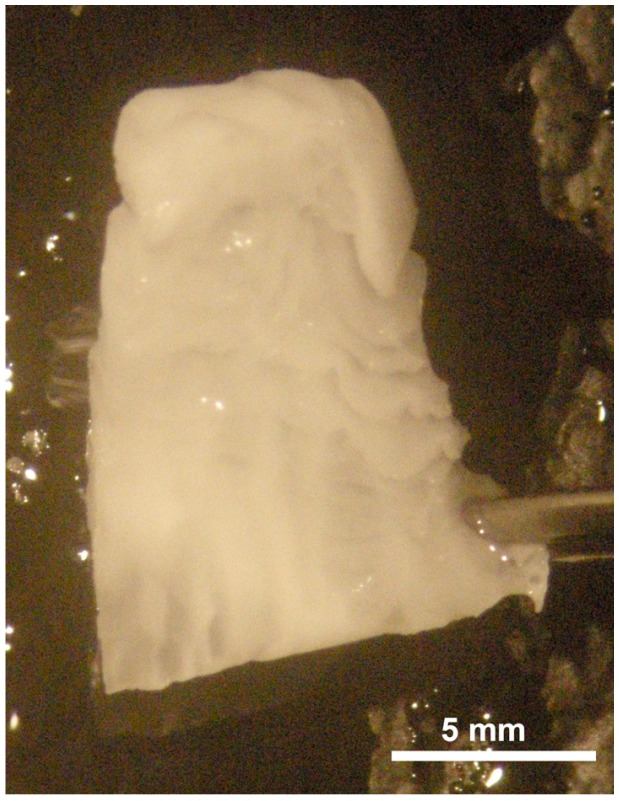
Light micrograph of 700 µm thick PCL (poly(ε-caprolactone)) electrospun-nanofiber scaffold.

A 700 µm thick electrospun PCL nanofibrous scaffold was manufactured to generate the tissue engineered bone graft (Figure 1). Electrospun nanofibers have an extremely high specific surface area, due to small fiber diameters, mimicking the collagen extracellular matrix [7].

Our aim was to investigate the capacity of osteoblasts cultivated as 3D microtissues to colonize the thick matrix and to lead to mineralization (bone formation). Thus, we followed *in vitro* the cell infiltration potency of osteoblasts (OB) microtissues seeded on the scaffold, by fluorescence confocal microscopy, and traced mineralization through Alizarin Red staining histology.

**Figure 2:**
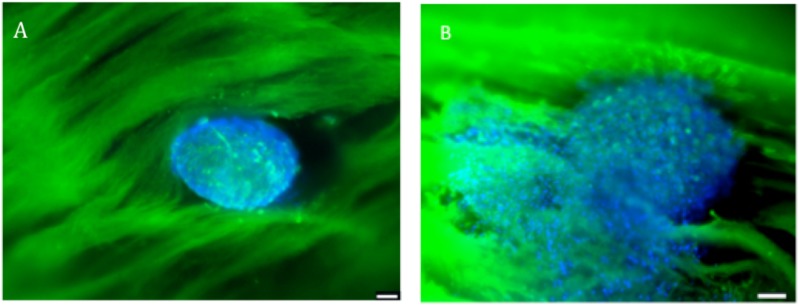
Fluorescence confocal microscopy visualization of human primary osteoblasts microtissues seeded onto the scaffold, and stained with DAPI (showing DNA) and PLL-FITC (showing the nanofibers). (**A**) Microtissue nesting in the scaffold at day 0; (**B**) Microtissue at day 3 of *in vitro* culture in the scaffold. Scale bar = 100 µm. *n* = 3.

After microtissue adherence onto the scaffold, cells began to spread after 3 days of culture, exhibiting osteoblast migration along the nanofibers (Figure 2).

**Figure 3:**
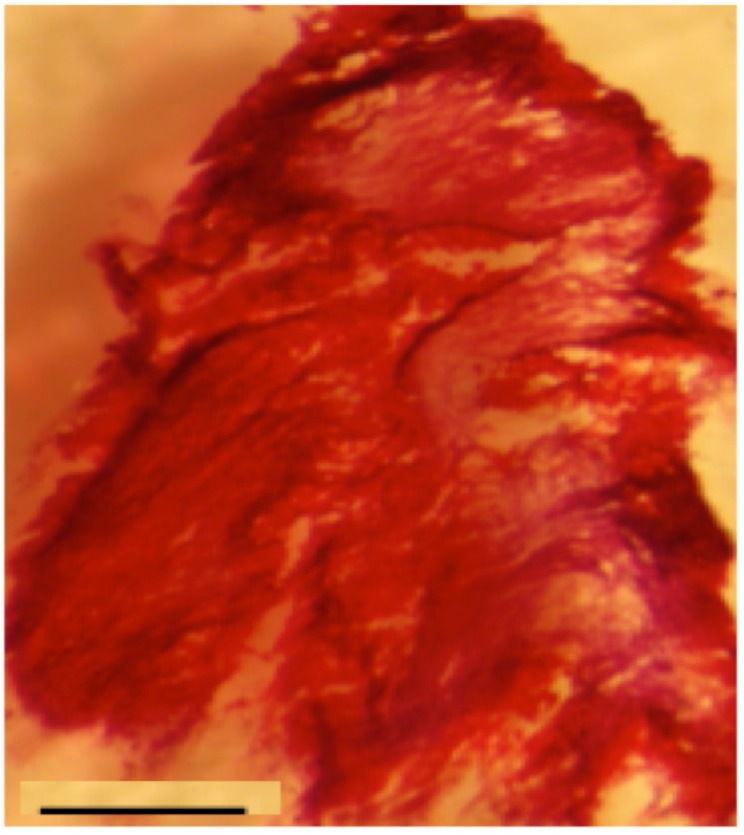
Alizarin Red staining of PCL scaffold seeded with Osteoblasts microtissues: *In vitro* bone induction and mineralization on the PCL electrospun nanofibers seeded with microtissues after 28 days of *in vitro* culture. Scale bar = 1 mm. *n* = 3.

Staining by Alizarin Red to check the formation of neo-calcified tissue revealed high bone formation within the core of the thick PCL scaffold seeded with OB microtissues after 28 days (Figure 3).

**Figure 4:**
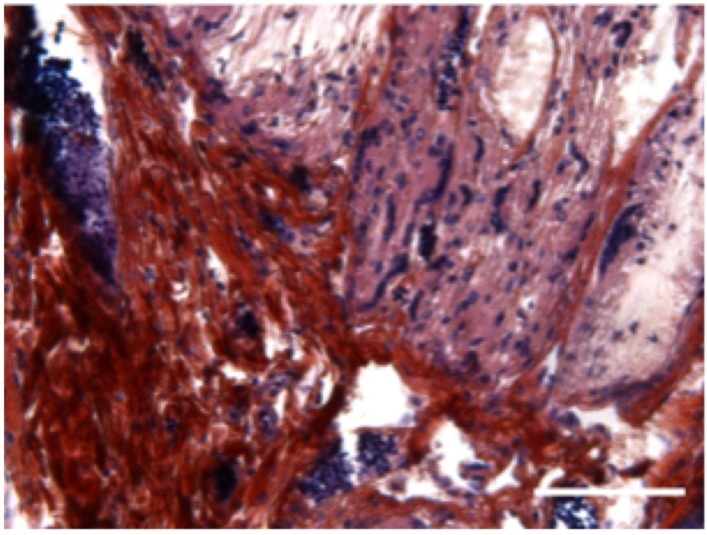
Subcutaneous implantation of hybrid 3D electrospun-nanofibrous implant in mice leads to bone formation: *In vivo* bone induction in PCL 3D scaffold with human primary osteoblasts microtissues 4 weeks after subcutaneous implantations in nude mice. Scale bar = 100 µm. *n* = 5 mice.

*In vivo*, subcutaneous implantation of the hybrid 3D implant was performed on nude mice. Implants were recovered after four weeks, cut in sections and stained with Mallory coloration. The histological sections clearly demonstrate that cells originating from the microtissues migrated and colonized the whole scaffold (Figure 4).

Here we demonstrate that combining two conceptual different tissue-engineering strategies, PCL nanofibers scaffolds (3D biomaterial) seeded with bone microtissues (3D cell aggregate), leads to bone tissue formation observed *in vitro* and *in vivo*, after subcutaneous implantation, without even any need of growth factors.

Some recent study has shown that the release of BMP-2 growth factor from capsules together with injection of embryonic stem cells without the presence of a hydrogel matrix did not induce bone regeneration, but it did when a scaffold was additionally implanted [8]. Recently, in our group, we have shown that by using the present thick 3D PCL nanofibrous scaffold, combined with single cells, the cell colonization and mineralization of the implant occur but without any bone induction in the core of the implant (submitted manuscript). Thus the implantation of this kind of scaffold is not enough in the case of large lesions, even with injection of single cells: To favor maturation of the tissue throughout the entire scaffold, the optimization of cell-cell-interactions (and not solely cell-scaffold interactions) as they prevail in natural tissues, is an important factor [3,9]. In consequence, the second strategy consisting in seeding cell microtissues in the scaffold appears most relevant.

## 3. Experimental Section

**Chemicals.** Poly(ε-caprolactone) (PCL), analytical grade (MW 80000), was obtained from Perstorp (Sweden). PCL was dissolved in a mixture of dichloromethane/dimethylformamide (DCM/DMF 40/60 *v*/*v*) at 27% w/v and was stirred overnight before use.

**Electrospinning.** A standard electrospinning set-up apparatus EC-DIG purchased from IME Technologies (Eindhoven, Netherlands) was used to fabricate the PCL 3D nanofibrous scaffolds. The PCL solution was poured into a 5 mL syringe and ejected through a 21G needle of 0.8 mm outer diameter at a flow rate of 1.2 mL/h via a programmable pump (ProSense). The electrospun jet was focused by the use of a poly(methyl methacrylate) (PMMA) 2.5 mm thick mask drilled with a hole (diameter of 25 mm) placed over the conductive collector. The collector was placed at a 16 cm distance from the needle. A voltage of +15 kV was applied on the needle, whereas −5 kV was applied on the collector during the electrospinning process.

**Cell culture.** Human primary osteoblasts were obtained from PromoCell (Heidelberg, Germany). Osteoblasts were cultured in a specific osteoblast growth medium together with complement and 50 U·mL^−1^ penicillin, 50 µg·mL^−1^ streptomycin, 2.5 µg·mL^−1^ amphotericin B. The cells were incubated at 37 °C in a humidified atmosphere of 5% CO_2_. When cells reached sub-confluence, they were harvested with trypsin and sub-cultured.

**Microtissue culture.** Osteoblasts were seeded in GravityPLUS™ plate (InSphero AG, Zürich, Switzerland) to produce microtissues. 1 × 10^4^ cells per microtissue were seeded in these plates and cultivated during 5 days. For bone formation, osteoblast microtissues were then seeded onto the PCL 3D scaffolds for *in vitro* studies and *in vivo* implantations.

***In vitro* analysis of mineralization using Alizarin Red S staining.** Alizarin Red S powder was dissolved in distilled water at a concentration of 2 g for 100 mL. The samples were incubated in the Alizarin Red solution for 20 min and then rinsed with distilled water several times. The samples were embedded in Tissue-Tek OCT™ Compound to be cut in sections (35 µm) with a cryostat (LEICA JUNG CM 3000). The sections were then observed under the optical microscope (LEICA DM 4000 B). Number of experiments *n* = 3.

**Confocal microscopy.** Human osteoblast single cells or microtissues were seeded onto PCL 3D electrospun scaffolds and cultivated for 1 and 21 days before fixation with PFA 4%. Then, cell nuclei were stained with DAPI and nanofibers were stained with PLL-FITC. Fluorescence microscopy was performed with a confocal microscope Zeiss LSM 700.

***In vivo* subcutaneous implantation in nude mice.** All procedures were designed in compliance with the recommendations of the European Union (2010/63/EU) for the care and use of laboratory animals. Ethics statement: Experiments followed current European Union regulations (Directive 2010/63/EU), and were performed according to authorized investigator N. Jessel (Director of the “Osteoarticular and Dental Regenerative Nanomedicine” Team), holder of a personal license from “Préfecture du Bas-Rhin” (No. 67-315), who oversaw experiments done on mice. All experiments were done in the “Animalerie Centrale de la Faculté de Médecine de Strasbourg” with the approval number: A 67-482-35 from the Veterinary Public Health Service of the “Préfecture du Bas-Rhin”, representing the French Ministry of Agriculture, Department of Veterinary Science. All surgery was performed under Ketamine and Xylazine anesthesia, and all efforts were made to minimize suffering. The study was run with Nude male mice (Crl: NIH-Foxn1^nu^ Charles River, France) 6 weeks of age. The mice were anesthetized with an intra-peritoneal injection of 100 mg/kg of ketamin (VIRBAC Santé Animale, Centravet, Italy) and 10 mg/kg of Xylazin (Rompun^®^ 2%, Centravet, Italy). Mice were anesthetized and implanted with a PCL 3D implant seeded with osteoblast microtissues. The samples were implanted between skin and muscles behind the mice ears. *n* = 5 mice were used. After 4 weeks of implantation, mice were sacrificed and the samples extracted for analysis.

**Histological analysis.** The implants were fixed with Bouin Hollande solution during 2 days. Then, they were dehydrated through a series of increasing ethanol concentrations, cleared with toluene and embedded in paraffin wax. Sections were cut at 7 µm using a sledge microtome and mounted on glass slides. After the removal of paraffin wax, sections of subcutaneous implants were stained using Mallory coloration during 2 days. Number of experiments *n* = 3.

## 4. Conclusions

Here we report the development and therapeutic impact of advanced bone implants combining nanostructured biomaterials with cell microtissues. We suggest reconsidering the current tissue-engineering approach, by seeding cell microtissues instead of single cells onto scaffolds. By using nanofibrous scaffolds, we believe that this strategy could lead to a new generation of higher-quality engineered tissues, with accelerated production times and potentially lower costs. This concept is not limited to bone tissue engineering and might operate also for other tissues such as skin tissue engineering.
