# Addendum: Keller, L.; *et al.* Integrating Microtissues in Nanofiber Scaffolds for Regenerative Nanomedicine. *Materials* 2015, *8*, 6863–6867

**DOI:** 10.3390/ma8125466

**Published:** 2015-12-03

**Authors:** Materials Editorial Office

**Affiliations:** MDPI AG, Klybeckstrasse 64, CH-4057 Basel, Switzerland

The *Materials* Editorial Office wishes to make the following erratum to this paper [1].

The affiliation number for the co-author Anne-Marie Musset in the pdf file should be changed from 1,2,3 to 2,3.

For any questions, please contact the editorial office of the journal or support@mdpi.com.
